# Drying Induced Impact on Composition and Oil Quality of Rosemary Herb, *Rosmarinus Officinalis* Linn

**DOI:** 10.3390/molecules25122830

**Published:** 2020-06-19

**Authors:** Hamdoon A. Mohammed, Mohsen S. Al-Omar, Salman A. A. Mohammed, Mohamed S. A. Aly, Abdulmalik N. A. Alsuqub, Riaz A. Khan

**Affiliations:** 1 Department of Medicinal Chemistry and Pharmacognosy, College of Pharmacy, Qassim University, Qassim 51452, Saudi Arabia; M.OMAR@qu.edu.sa; 2 Department of Pharmacognosy, Faculty of Pharmacy, Al-Azhar University, Cairo 11371, Egypt; 3 Medicinal Chemistry and Pharmacognosy Department, Faculty of Pharmacy, JUST, Irbid 22110, Jordan; 4 Department of Pharmacology and Toxicology, College of Pharmacy, Qassim University, Qassim 51452, Saudi Arabia; M.AZMI@qu.edu.sa; 5 Hospital of the Police Academy, Nasr City, Cairo 11765, Egypt; mohamedshawky1974@yahoo.com; 6 Pharm D Student, College of Pharmacy, Qassim University, Qassim 51452, Saudi Arabia; aabdulmaliknn@gmail.com

**Keywords:** *Rosmarinus officinalis*, volatile oils, natural drying, antioxidant activity, compositional ratio

## Abstract

The natural drying of *Rosmarinus officinalis* Linn. herbs severely affects its volatile oil quality and yields, which is reported here for the first time. The oils obtained through hydrodistillation from fresh, one, two, and three-weeks dried herbs were analyzed by gas chromatography–mass spectroscopy (GC–MS) and gas chromatography–flame ionization detector (GC-FID), and the yields were 198 ± 3.45, 168.7 ± 5.11, and 97.8 ± 1.27 mg, respectively, as compared to the internal referral standard of 327 ± 5.91 mg yield of the one-week dried herbs’ oil. Camphor, the major constituent, significantly depleted from 20.96% to 13.84%, while bornyl acetate yields increased from 1.42% to 12.46% (*p* values < 0.0001) in three-weeks drying, reflecting the redox processes undergoing within the oil during drying. Several constituents (25) were found in one-week dried herbs’ oil as compared to the fresh, two-, and three-weeks oils, which consisted of 23, 19, and 14 constituents, respectively, leading to the recommendation of the one-week drying of the herb for maximum oil yield. The DPPH (2, 2-diphenyl-1-picryl-hydrazyl) reactivity was highest for the two- and three-weeks dried herb-based oils, followed by the one-week dried- and fresh-herb-based oils (*p* < 0.0001), again indicating major chemical changes during herbs’ dryings, affecting the free-radical scavenging capacity of these batches of oils obtained after different drying times.

## 1. Introduction

The *Rosmarinus officinalis* Linn., commonly known as rosemary, is an evergreen worldwide cultivated herbaceous plant belonging to the family Lamiaceae [1]. Rosemary has economic values owing to its medicinal properties and culinary usage, and it is frequently used in confectionery, perfumery [2,3], and food preservatives, especially meat [4]. Commercially, rosemary and its oil are highly-prized [5]. The plant is also available as dried herbs, with herbalists selling the aromatic and medicinal plants together with its oil in the community pharmacy. The herb acts as a remedy in treating several day-to-day common health-problems such as stomach ache, common cold, and cough [6]. The plant is still in use in folk medicine for the symptomatic relief of inflammation of joints, as a diuretic, and in the management of diabetes and cardiovascular disorders [3]. Besides, the plant’s volatile oil is traditionally used as a pain-killer, carminative, antispasmodic, and for treatments of renal colic. The herb essential oils and extracts have also been used as cholagogue, expectorant, and hair growth [7]. The volatile and non-volatile constituents cause to the medicinal effects of *R. officinalis* [8,9]. The rosmarinic acid, and diterpenes such as carnosic acid, carnosol, and rosmanol, in addition to other non-volatile triterpenic constituents, with promising broad-spectrum biological activities, as betulinic and ursolic acids are found in the herb [3,10]. The earlier investigations also reported significant differences in the herb’s volatile oil constituents following the particular habitat of the plant variety, confirming the geographically-distributed different genotypes or the chemotypical variations [11,12,13,14]. The similar phenotypical plant pool of rosemary herbs provides altered oil yields with different proportions and the presence of constituents in oils, purely owing to different conditions of extractions, e.g., hydro- and steam-distillations, supercritical fluid, supercritical CO_2_ extractions, and water microwave-assisted hydrodistillation [15,16,17,18,19]. The camphor and 1,8-cineole, the two major volatile oil constituents of rosemary herbs, are present in all plant varieties of chemo/pheno-typic, and genotypic plant variations. The yields of these constituents were significantly affected by the change of extraction methods within the similar pheno/chemo-typic plants. The camphor yields percentage increased from 12.6% to 19.7% in water distillation over the steam-distillation process, while the proportions of 1,8-cineole obtained from the steam-distillation process decreased from 52.4% to 31.9% with the change of oil extraction methodology from steam- to hydro-distillation [17]. 

The initial drying is an essential process for medicinal plants for fixating and preserving their constituents [20]. The natural drying processes refer to plant materials drying by sunlight or in the shade under existing environmental conditions [20,21]. Drying effects are recorded for their impact on oil yields, constituents, and the compositional build-up of the oil constituents, along with the medicinal properties [21,22,23,24]. The effects of various drying methodologies over volatile oil yields are an ostensibly common phenomenon. For instance, for fresh herbs and their microwave-drying, vacuum oven-drying, hot air drying, and freeze-drying, affect oil yields, the number of constituents, and quality, and they have shown substantial differences among the contents in up and down trends of yields in various drying procedures [15,16,23,24,25,26]. The present study adds information about the natural drying of the herbs, as practiced in herbalist drying protocol, and at homes before fresh rosemary herb’s storage for prolonged use or for the extraction of the oil for various medicinal and culinary uses. This study investigated drying and extended drying periods’ effects on oil yields and oil’s constituents, together with their compositional ratios in the oil. The study also analyzed the antioxidant property of the oil as an indicator of its quality. 

Furthermore, no studies are available on rosemary oil on the effects of drying-led chemical transformations, including redox reactions of the oil constituents concerning air-exposure and other harsh conditions of moisture and light on the drying of fresh rosemary herbs. The present investigation analyzed the aspects of the oil yields and quality, and it attempted to correlate the presence and proportions of different oil constituents over different weeks of dried and fresh rosemary herbs. 

## 2. Results and Discussion

The extended natural drying of rosemary herbs at room temperature and under air availability in shade conditions significantly affected the herbs’ oil yield and quality. The differences in oils’ constituents and their compositional ratios in different drying batches are reported. The overall antioxidant activity of different batches of oils measured as DPPH radical scavenging activity also showed remarkable differences.

### 2.1. Rosemary Oil Productivity in Different Drying Periods

The drying data exhibited the weights of rosemary herbs after different periods of natural drying in the shade at room temperature. One week of drying showed a substantial reduction (65%) in the weights of the initial fresh herb from 200 g to 70 g. However, the weight reductions between the two- and the three-week dryings were insignificant (Table 1). The amount of volatile oils obtained from the fresh plant (200 g) by hydrodistillation was 198 ± 3.45 mg, as compared to 327 ± 5.91, 168.7 ± 5.11, and 97.8 ± 1.27 mg of the volatile oils obtained from the dried herbs of 70, 68, and 68 g weights after one, two, and three weeks of natural dryings, respectively. The oil weight reduction between the first and second week was about 48.4%, while for the third week, it was ~22% of losses as compared to the second week dried herbs (Table 1). Moreover, the notable increases in the volatile oil yields after one-week of the natural shade-drying of the herb as compared to the fresh, two-, and three-weeks dried herbs oils were ~40%, 48%, and 70%, respectively, thus indicating the economic benefits of the one-week drying procedure for the best production yields of the oils from rosemary. Based on the observations, the one-week drying procedure could be recommended for small and large-scale productions of rosemary’s volatile oils through the hydrodistillation process.

### 2.2. Rosemary Oil Constituents in Different Drying Periods

**Figure 1:**
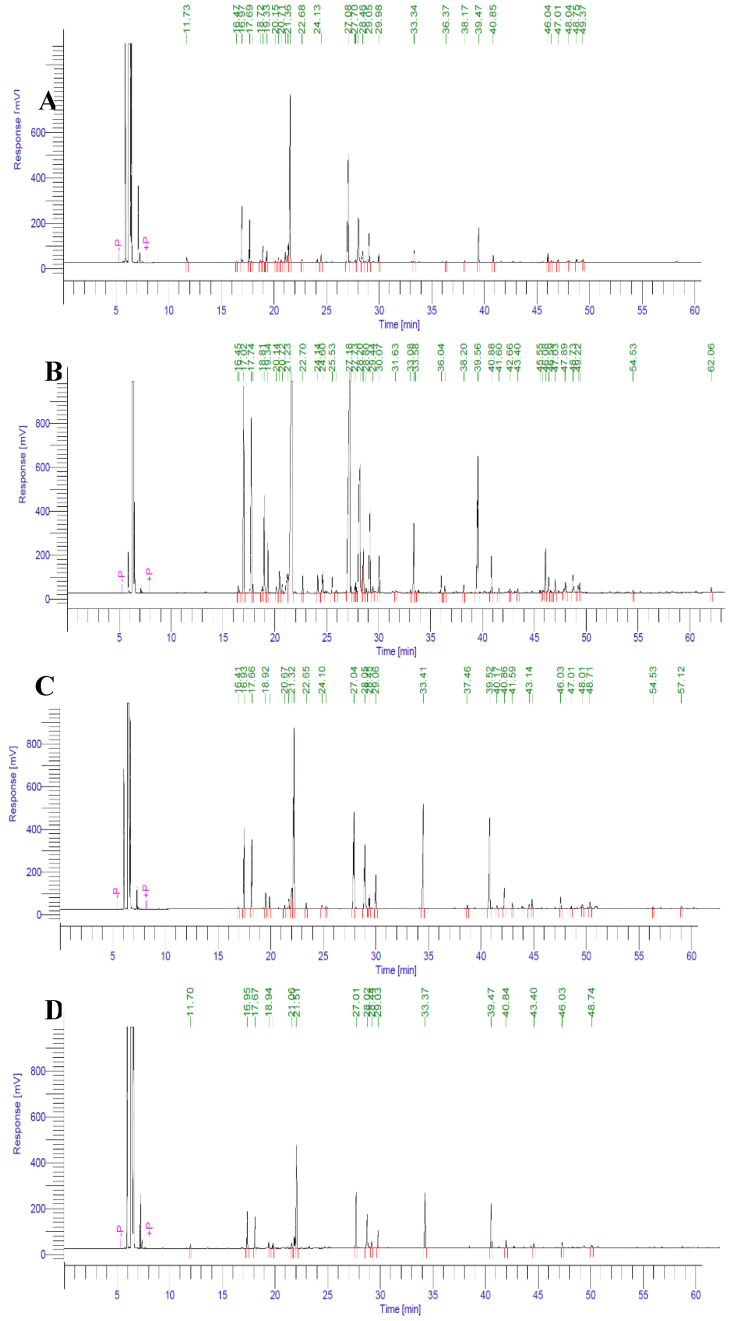
The gas chromatography (GC) chromatograms of rosemary’s volatile oils obtained from fresh (**A**), one-week dried (**B**), two-weeks dried (**C**), and three-weeks dried (**D**) herbs showed major differences in the number of peaks and their relative intensities in rosemary oil batches.

None of the major oil constituents, namely eucalyptol (1,8-cineole), camphor, and α-terpineol, were lost in any of the four samples of the herbs, i.e., the fresh herbs of the one-, two-, and three-weeks dried herbs, as observed by gas chromatography–mass spectroscopy (GC–MS) analyses. However, minor constituents’ ratios varied significantly in differently time-dried oil samples (Figure 1 and Table 2). The occurrence of oils’ constituents and their compositional ratios differed among all the oils obtained after different time-periods of drying. However, the compositional ratios of the oils differed less vigorously than the variation in numbers of the oils’ constituents in different batches of oils obtained from differently-timed dried rosemary herbs (Table 2). Borneol (*RI* 1165), isobornyl formate (*RI* 1237), and piperitenone (*RI* 1342) were absent in the fresh, two-, and three-weeks dried herbs’-based oils. The one-week dried herb oil was only devoid of terpinolene (*RI* 1090), which was 1.12–1.52% (in a statistically insignificant ratio; *p* = 0.2009) of the oil components in fresh, two, and three-weeks dried herbs’ oils. The fresh herb’s oil contained fewer constituents (23) compared with the oil that was obtained from the one-week dried herbs samples (25 constituents) due to the extraction shortfall and to the presence of excessive water in the fresh herb at the hydrodistillation time. The lowered extractability of the lipophilic constituents of oil in the presence of excess water in the fresh herbs favored the hydrophilic over the hydrophobic oily components, thus leading to lesser oil yields; see Table 1 and Table 2. The evidence that the oil yield from the fresh and one week dried herbs significantly differed additionally supports the notion. Quantitatively, the oil yields difference in fresh (198 ± 3.45 mg) and two weeks dried herbs (168 ± 5.11 mg) was ~30 mg. In comparison, the three-week drying of the herbs yielded the least oil (97.8 ± 1.27 mg) due to the excessive drying and gradual evaporation of the volatiles from the herbs under the three-weeks of drying, given that all the dryings were performed under similar conditions of temperature, atmospheric pressure, air current availability, and shade throughout the experiment.

Among all the components of the oils, 1, 8-cineole, camphor, and α-terpineol—together with β-caryophyllene, camphene, β-pinene, citronellol, bornyl acetate, and linalool—were the major components in all the batches of oils obtained from fresh and differently-timed dried herbs. Additionally, 1,8-cineole, camphor, and α-terpineol were the major components that constituted about 28.59%, 20.96%, and 8.92% of the fresh herbs-based oil and 24.12%, 19.64%, and 9.01% of the one-week dried herbs extracted oils, respectively (significant differences of these constituents in the fresh and one-week dried herbs showed only for 1,8-cineole with *p* = 0.0006, Table 2). The componential ratio of these constituents changed drastically, and the camphor components in the three-week dried herbs reduced in comparison to the fresh herbs oil by about 7%, a substantial change (*p* < 0.0001) with a significant variation in yields.

The observation included major decreases in yields and the number of oil constituents for the oils obtained from the two- and three-weeks naturally dried samples of the herbs, wherein only 19 and 14 components were found in the obtained oil samples, respectively, as compared with the 26 compounds in the one week dried herbs-based oil (Figure 1 and Table 2). The yields of camphene, β-pinene, α-terpineol, bornyl acetate, β-caryophyllene, and d-germacrene increased in the two- and three-weeks dried herbs-based volatile oils as compared to the fresh and one-week dried herbs-based oils; however, significant variations were shown only for bornyl acetate, β-caryophyllene, and d-germacrene with *p* values of <0.0001, 0.0001, and 0.0263, respectively, compared to fresh herbs-based oil. Moreover, the fresh herb oil analysis indicated the presence of higher concentrations of 1,8-cineole (28.59%), camphor (20.96%), citronellol (3.98%), linalool (3.29%), and verbenone (2.04%), which were common to all dried herbs, but only 1,8-cineole showed significant differences (*p* = 0.0006) between the fresh herbs and other batches. The one-week dried herbs’ oil also contained camphene (7.19%), α-terpinene (2.99%), and p-cymene (1.33%) as the highest concentration constituents in comparison to all other oil batches, although only α-terpinene showed significant differences (*p* < 0.0001). The borneol, isobornyl formate, and piperitone were only present in the one-week dried herbs-based oil under 0.5% concentrations, providing exclusive fragrance, yield, and maximum components. Again, the presence of myrcene, α- and β-phellandrene, and isobornyl acetate were found only in the fresh and one-week-based oils. Meanwhile, the two- and three-week dried herbs showed different compositions of their respective oils in terms of the foregoing constituents. The majority compounds of the two- and three-weeks dried oils were 1,8-cineole, camphor, bornyl acetate, and β caryophyllene (Figure 1 and Table 2). These fluctuations indicated that the volatile profiles of these oils were different according to time drying (Illustration Figure for the rosemary oils’ componential percentages in fresh and dried samples is available in the Supplementary Materials).

### 2.3. Possible Chemical-Biotransformations in Oil Constituents during Drying Periods

**Figure 2:**
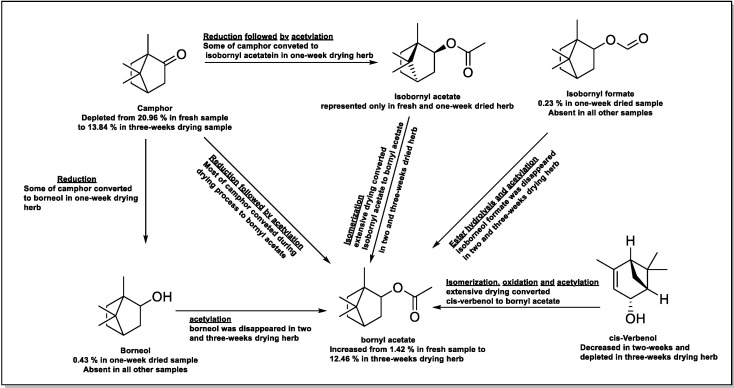
A plausible pathway for envisaged biotransformations within rosemary oil during the three-weeks long drying period.

The up and down changes in the componential ratios of the oil constituents of all the dried herbs concerning the fresh herbs-based oil, as shown in Table 2 in the absence of any chemical or physical treatments of the herbs batches, can be attributed to biotransformations of various oil constituents during the dryings, especially in the extended two- and three-weeks dryings of the herbs. Volatile oil components are the most sensitive plant constituents to plant-drying programs [27,28,29]. Furthermore, the chemical reactivity and medicinal properties of any particular plant/herb may vary because of these factors of drying methods and drying periods as intended for oil procurement [21,27,28] from any of the extraction procedures. Biochemical oxidation, reduction, and acetylation are the major chemical transformations occurring during the drying processes. The biotransformation reactions seem to be undertaken during the drying times for the rosemary herbs (Figure 2). It is a known fact that borneol is oxidized by borneol dehydrogenase to camphor [30], while camphor gets converted to its reduced form, borneol [31]. The bioconversions of camphor to bornyl acetate and isobornyl acetate; borneol oxidation to bornyl acetate; and *cis*-verbenol, and isobornyl formate oxidations to bornyl acetate are some of the examples of biotransformations. Accordingly, the steady increase in the percentages of bornyl acetate at the outset may be linked to a decrease in the percentage of camphor in different oil samples with an increase in the drying times of the herbs (Table 2). Furthermore, some of the identified components in fresh and one-week dried rosemary oil samples were absent in the two- and three-weeks-dried samples. The isobornyl acetate, myrcene, and α- and β-phellandrene could be bio-converted to other components due to their absence in these periods dried herbs’ oils. A closer look at the oil yields and their componential ratios between the fresh herbs and one-week dried herbs-based oils exhibited the presence of major components in nearly comparable yields with a significant decrease in 1,8-cineole and camphor yields proportions, while other constituents were either at par or at increased levels in their yields in the one-week dried herbs-based oil. The relationship of the componential yields and biotransformation with the interplay of drying periods on the oil contents and quality was a complex relationship. Nonetheless, the significant increase of bornyl acetate from 1.24% and 2.34% in the oil obtained from fresh and one-week dried herbs to 12.25% and 12.46% in the oil obtained after the two- and three-weeks drying periods was remarkable and was accompanied by the significant steady-decreasing order of camphor with the extending drying of the herb (Figure 2 and Table 2). These marked changes in the oil batch constituents pointed to the definite biotransformation taking place during the drying hours. These ongoing observations also led to the conclusion that the yields and componential ratios of the oil constituents in the present case of rosemary oils were dependent on the drying periods whereby all other factors of temperature, mode of heating, condensation apparatus cooling gradient, the duration of extraction, and the presence of water in the extraction assembly were kept same.

### 2.4. Antioxidant/Free Radical Scavenging-Based Quality of the Oil Samples

**Figure 3:**
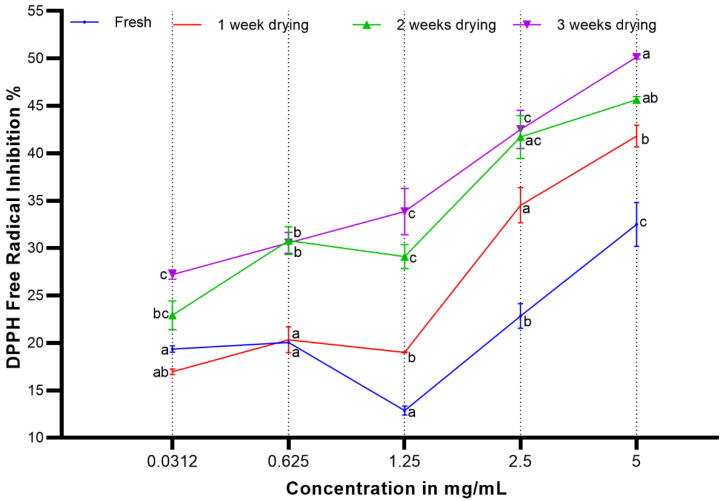
The antioxidant activity levels of rosemary oils agent DPPH-stable free radical tests for the fresh herbs (zero dryings), one-week, two-weeks, and the three-weeks dried herbs oil’s DPPH reactivity. The statistical significance of the two-way ANOVA was *p* < 0.0001. Tukey’s multiple comparisons were then performed for all the different concentrations. Different letters within the same concentration denote significant differences among the groups according to Tukey’s test (*p* < 0.01).

There are several reports on the antioxidant activity of rosemary oil gathered by various methods [32,33,34]. The antioxidant DPPH-based reactive values were used as a comparative factor for estimating the quality of oils obtained through various drying periods with a changed spectrum of its componential constituents. The DPPH-reactivity of the oils obtained from rosemary samples after various drying periods, together with the fresh herbs, indicated the oil compositions-based anti-oxidant behavior due to the presence and absence of the various constituents in the different batches of oils. The results shown in Figure 3 indicate the moderate DPPH-scavenging activity of the rosemary oil. They revealed that the antioxidant activity of the *Rosmarinus officinalis* volatile oil increased proportionally with the increase in the natural drying periods of the herb, which is an indication of the accumulation of antioxidant products through biotransformation. Moreover, the anti-oxidant activity for the three-weeks dried herb-based oil did not significantly differ compared to the two-week dried herb-based oil at all the measured concentrations with comparable antioxidant activity at 0.625 and 2.5 mg/mL, respectively (Figure 3). Likewise, the comparable anti-oxidant activity of the fresh and one-week dried herbs-based oils at 0.0312 and 0.625 mg/mL, respectively, are also noteworthy. The highest anti-oxidant activities of all the batches of oils were at 32%, 41%, 46%, and 51% inhibitions (*p* < 0.0001) of the DPPH radicals at 5 mg/mL concentrations for the fresh and one-, two-, and three-week dried herb-based oils, respectively. Again, the DPPH test responses from the fresh and one-week dried herbs’ oils were altogether different and at significantly (*p* < 0.0001) lower levels than the two- and three-weeks dried herbs-based oils. It is noteworthy that the significant compositional differences between the two and three weeks oils and the fresh and one-week dried herbs-based oils were in the oil batches containing a major presence of bornyl acetate in two- and three-weeks dried herbs-based oils (fresh, one, two, and three weeks oils: 1.42%, 2.24%, 12.25%, and 12.46%). In comparison, the ratio of the yields of the camphor decreased in the two- and three-weeks dried herbs-based oils (fresh, one, two, and three weeks oils: 20.96%, 19.64%, 12.09%, and 13.84%), which seems to be an apparent reason for higher anti-oxidant activity of the two- and three-weeks dried herbs-based oils.

The dose-dependent DPPH reactivity changes are noteworthy and were concluded based on the enhanced presence of particular constituents after the analysis of the componential ratios in different batches of oils (Table 2). The significantly higher levels of DPPH reactivity at 5 mg/mL (*p* < 0.0001) of the two- and three-weeks dried herbs’ oils, and the reactivity at equal levels at lower concentrations of 0.0312 and 0.625 mg/mL for the fresh and one-week dried herbs-based oils, respectively, can be attributed to the presence of structurally-similar constituents or common chemical class compounds, although the synergistic action of other constituents and other chemical class compounds cannot be ruled out. Moreover, the abundance of terpenoids with hydrocarbons and C, C-double bonds (C=C) is one apparent reason for the significant higher DPPH-reactivity of the two- and three-weeks dried herbs-based oils, as reflected in the presence of the oil constituents and compositions. The three-week dried herb oil also contained various terpenic constituents of alcoholic, ketonic, and aldehyde nature in reduced proportions in comparison to all the other oils. The fresh and one-week dried herbs-based oils also contained this category of hydrocarbons, terpenes, and C=C containing compounds as oil constituents at 44.5% and 48%, respectively. It is noteworthy that hydrocarbons and C,C-double bond (C=C) constituents are more susceptible to DPPH reactivity [35,36], and the three-weeks oil clearly showed higher levels of DPPH reactivity, followed by the two-weeks dried herbs-based oil (Figure 3).

## 3. Materials and Methods 

### 3.1. Plant Materials Collection

Approximately one-year-old *Rosmarinus officinalis* herbs were collected in February 2019 from the gardens of the Ministry of Agriculture in Qassim, Kingdom of Saudi Arabia. Local botanists identified the plant material, and a specimen was preserved. 

### 3.2. Plant Preparation and Distillation Method

Fresh rosemary herbs were equally divided into twelve weight groups of 200 g each. The first group (3 × 200 g, fresh herbs) was cut into small pieces by scissors and subjected to hydrodistillation using Clevenger’s apparatus in three cycles (200 g each) to isolate its volatile oil contents. The second group’s herbs materials (3 × 200 g), obtained after one week of natural drying (at room temperature and natural atmospheric pressure under standard laboratory conditions) in the shade were also cut into smaller pieces and subjected to hydrodistillation. Likewise, the third (3 × 200 g) and fourth (3 × 200 g) groups’ herbs materials were also subjected to hydrodistillation after the similar drying conditions for two and three weeks, respectively. The hydrodistillation procedures were conducted for 5 h for each cycle, and the results summarized in Table 1. All the obtained oils were dried by passing through anhydrous sodium sulfate, weighed, and stored at −20 °C. 

### 3.3. Gas Chromatography–Mass Spectroscopy (GC–MS) Analyses

The GC–MS analysis was performed on Shimadzu GCMS-QP 2010 (Kyoto, Japan) equipped with an Rtx-5MS capillary column (30 m × 0.25 mm i.d. × 0.25 µm film thickness) (Restek, Bellefonte, PA, USA) according to the reported procedure [37]. The oven temperature was kept at 50 °C for 3 min (isothermal) and programmed to 300 °C at 5 °C/min, and then it was kept constant at 300 °C for 10 min (isothermal); the injector temperature was 280 °C. The helium was used as a carrier gas with a constant flow rate set at 1.37 mL/min. Diluted samples (1% *v*/*v*) were injected with a split ratio of 15:1, and the injected volume was one μL. The MS operating parameters were as follows: interface temperature: 280 °C; ion source temperature: 220 °C; and EI mode: 70 eV; and scan range: 35–500 amu.

### 3.4. Gas Chromatography-Flame Ionization Detector (GC-FID) Analyses

The GC analysis was performed on a Perkin Elmer Auto System XL equipped with a flame ionization detector (FID). A fused silica capillary column ZB5 (60 m × 0.32 mm i.d. × 0.25 μm film thickness) was used. The oven temperature was maintained initially at 50 °C and programmed from 50 to 240 °C at a rate of 3 °C/min. The helium was used as the carrier gas at a flow rate of 1.1 mL/min. The injector and detector temperatures were 220 and 250 °C, respectively. 

### 3.5. Identifications of the Essential Oil Constituents

The essential oil constituents obtained from fresh rosemary and naturally dried samples were identified based on the experimental retention index (*RI*) calculated in comparison to a series of n-alkenes (C_8_–C_40_) and a retention index obtained from the literature under similar GC experimental conditions. The identification of the compounds was carried out based on retention time, mass fragmentation patterns, and mass spectral libraries (National Institute of Standards and Technology (NIST-11) and Wiley Database). The relative percentages of the constituents was calculated from the area under the peak obtained from the GC-FID chromatogram.

### 3.6. Determination of the Antioxidant Activity of Oil Samples by DPPH

The antioxidant activity of different oil batches was determined by the DPPH method [38]. Quercetin was used as a standard. Briefly, 0.5 mL of the rosemary’s volatile oils, in a concentration ranging from 5 to 0.0312 mg, was added to 1.5 mL of a DPPH–methanol solution at a concentration of 100 µM (quercetin was used as a standard in the same concentration [39,40]). The mixtures were then vortexed and set aside in the dark for 30 min before being measured at the 517 nm wavelength by a UV–VIS spectrophotometer. The inhibition of free radical DPPH (I%) was calculated by the formula: I% = (A_blank_−A_sample_)/A_blank_ × 100. The independent measurements were conducted in triplicate, and the standard error was calculated.

### 3.7. Statistical Analyses

The data are expressed as mean ± standard error of the mean (SE). The differences among groups were analyzed using a one-way ANOVA after the extraction of all 26 constituents versus the concurrent constituents present in each of the oil sample. Similarly, differences among groups was analyzed using a two-way ANOVA after the antioxidant test was performed. A post hoc test was performed using Tukey’s multi-group comparison on GraphPad Prism 8.0.2. The significance value was set at *p* < 0.05.

## 4. Conclusions

The biochemical transformations involving oxidation, reduction, and acetylation reactions were apparent in different oil samples obtained from the rosemary herbs that were naturally dried for different amounts of weeks. The presence of various constituents, their ratios in the corresponding oils, and their absence in certain oil batches from different drying slots of herbs, as represented in Figure 2 and Table 2, supported the contention. The results revealed that the best volatile oil yield by the hydrodistillation procedure of oil extractions with the majority of oil constituents present and comprising the 1,8-cineole, camphor, and camphene ingredients in higher ratios could be obtained after the first week of rosemary herbs’ shade-drying under natural conditions. The one-week dried herbs-based oil contained these three major constituents in 50.9% of the total oil compositions, while the fresh herbs’ oil contained these components of the oil at 55.9%. The same constituents in two and three week dryings-based oils were at 41.4% and 44.8%, respectively. The obtained oil was found to be closest in composition to the fresh herbs-based oil. Hence, we recommend a one-week natural, shade-based drying of the rosemary herbs for higher yields of the volatile oil at both industrial and small scales. Moreover, the antioxidant properties of the one-week dried oil were also at their optimum at higher concentrations of 5 mg/mL of the DPPH-based tests, although two- and three-weeks dried oil showed the best antioxidant values and can also be safely recommended as a traditional way of meat preservation, as has been practiced by several cultures. Though rosemary’s antioxidant activity has been established and is employed in many aliments, it’s claimed CVS, anti-diabetic, anti-inflammation, and anti-cancer bioactivities need independent confirmations.

## Figures and Tables

**Table 1:** Effects of natural drying on weights and oil yields from the rosemary herbs.

Period of Natural Drying	Weight of the Fresh Herbs (Grams)	Weight of Herbs at Distillation(Grams)	% Weight Loss of Herb	Volatile Oil Yields (Milligrams) ^1^	% Weight Loss of Oil ^2^
**Fresh**	200	200	0%	198 ± 3.45	39.44%
**One week**	200	70	65%	327 ± 5.91	100% ^2^
**Two weeks**	200	68	66%	168.7 ± 5.11	48.40%
**Three weeks**	200	68	66%	97.8 ± 1.27	70.09%

^1^ Volatile oil yields were calculated from three distillation procedures for each plant sample ± standard deviation. ^2^% weight loss of oil in comparison to the oil obtained from 1-week dried herbs.

**Table 2:** Rosemary oil components and their componential percentages in the fresh and dried herbs’ oil samples.

Serial No.	Components	Retention Index *(RI)*		The Percentage of the Total Area in GC–MS Analyses			
			One-Way ANOVA (*p*-Value)	Fresh Herbs	One Week Drying	Two Weeks Dryings	Three Weeks Dryings
**1.**	α-Pinene	932	0.0066 ***	0.14a	0.18b	0.15a	-
**2.**	Camphene	943	0.4512	6.42	7.19	6.82	6.99
**3.**	β-Pinene	973	0.4884	5.10	5.90	5.90	5.98
**4.**	Myrcene	990	-	0.19	0.20	-	-
**5.**	α-Phellandrene	1004	-	0.34	0.28	-	-
**6.**	α-Terpinene	1016	<0.0001 ***	2.07 a	2.99b	1.34c	1.83a
**7.**	p-Cymene	1025	0.4616	1.24	1.33	0.99	0.99
**8.**	1,8-Cineole (Eucalyptol)	1030	0.0006 ***	28.59a	24.12b	22.56cb	24.04db
**9.**	β-Phellandrene	1031	-	0.18	0.20	-	-
**10.**	Terpinolene	1090	0.2009	1.52		1.12	1.17
**11.**	p-Cymenene	1091	0.8203	0.34	0.46	0.47	-
**12.**	Linalool	1102	0.0019 **	3.29a	1.52b	3.01a	3.08a
**13.**	α-Campholenal	1127	0.0002 ***	0.94a	0.81a	0.22b	-
**14.**	cis-Verbenol	1142	0.3993	0.46	0.53	0.34	-
**15.**	Camphor	1145	<0.0001 ***	20.96a	19.64a	12.09b	13.84c
**16.**	Borneol	1165	-	-	0.43	-	-
**17.**	α-Terpineol	1193	0.8816	8.92	9.01	8.99	9.61
**18.**	Verbenone	1215	0.0763	2.04	1.30	1.34	1.47
**19.**	Citronellol	1227	0.9956	3.98	3.89	3.58	3.86
**20.**	Isobornyl formate	1237	-	-	0.23	-	-
**21.**	Isobornyl acetate	1285	-	1.13	1.28	-	-
**22.**	Bornyl acetate	1292	<0.0001 ***	1.42a	2.34a	12.25b	12.46b
**23.**	Piperitenone	1342	-	-	0.47	-	-
**24.**	β-Caryophyllene	1427	0.0001 ***	4.62a	6.21a	11.09b	9.91b
**25.**	Germacrene-D	1490	0.0263	0.90a	1.12	1.88b	1.71
**26.**	epi-α-bisabolol	1686	0.5035	0.26	0.38	0.49	-
**27.**	Total percentage of constituents accounted from GC–MS			95.05	92.01	94.63	96.94
**28.**	Total identified number of constituents			23	25	19	14
**29.**	The projected weight percentage of constituents ^1^			88.46	96.15	73.07	53.85

^1^ The statistically projected yields are based upon extractions of all 26 constituents versus the concurrent constituents present in each of the oil samples. Statistical significance of an ANOVA: ** *p* < 0.01; *** *p* < 0.001. Tukey’s multiple comparisons were performed for the constituents that were statistically significant using a one-way ANOVA. Different letters within the same constituent row denoted significant differences between the time points, according to Tukey’s test (*p* < 0.05).

## Supplementary Materials

The following are available online, Figure S1: Rosemary oils’ componential percentages in fresh and dried samples.

## References

[1] Bozin B., Mimica-Dukic N., Samojlik I., Jovin E. (2007). Antimicrobial and antioxidant properties of rosemary and sage (*Rosmarinus officinalis* L. and *Salvia officinalis* L., Lamiaceae) essential oils. J. Agric. Food Chem..

[2] Small E. (2006). Culinary Herbs.

[3] Ribeiro-Santos R., Carvalho-Costa D., Cavaleiro C., Costa H.S., Albuquerque T.G., Castilho M.C., Ramos F., Melo N.R., Sanches-Silva A. (2015). A novel insight on an ancient aromatic plant: The rosemary (*Rosmarinus officinalis* L.). Trends Food Sci. Technol..

[4] Nieto G., Díaz P., Bañón S., Garrido M.D. (2010). Dietary administration of ewe diets with a distillate from rosemary leaves (*Rosmarinus officinalis* L.): Influence on lamb meat quality. Meat Sci..

[5] Elmhalli F., Garboui S.S., Borg-Karlson A.-K., Mozūraitis R., Baldauf S.L., Grandi G. (2019). The repellency and toxicity effects of essential oils from the Libyan plants *Salvadora persica* and *Rosmarinus officinalis* against nymphs of Ixodes ricinus. Exp. Appl. Acarol..

[6] Trotter R.T. (1981). Folk remedies as indicators of common illnesses: Examples from the United States-Mexico border. J. Ethnopharmacol..

[7] Hassani F.V., Shirani K., Hosseinzadeh H. (2016). Rosemary (*Rosmarinus officinalis*) as a potential therapeutic plant in metabolic syndrome: A review. Naunyn. Schmiedebergs. Arch. Pharmacol..

[8] Moreno S., Ojeda Sana A.M., Gaya M., Barni M.V., Castro O.A., van Baren C., El-Samragy Y. (2012). Rosemary compounds as nutraceutical health products. Food Additives.

[9] Fahim F., Esmat A., Fadel H., Hassan K. (1999). Allied studies on the effect of *Rosmarinus officinalis* L. on experimental hepatotoxicity and mutagenesis. Int. J. Food Sci. Nutr..

[10] Ho C.-T., Ferraro T., Chen Q., Rosen R.T., Huang M.-T., Ho C.-T., Osawa T., Huang M.-T., Rosen R.T. (1994). Phytochemicals in teas and rosemary and their cancer-preventive properties. Food Phytochemicals for Cancer Prevention II.

[11] Baratta M.T., Dorman H.J.D., Deans S.G., Biondi D.M., Ruberto G. (1998). Chemical composition, antimicrobial and antioxidative activity of laurel, sage, rosemary, oregano and coriander essential oils. J. Essent. Oil Res..

[12] Jamshidi R., Afzali Z., Afzali D. (2009). Chemical composition of hydrodistillation essential oil of rosemary in different origins in Iran and comparison with other countries. Am. J. Agric. Environ. Sci..

[13] Özcan M.M., Chalchat J.-C. (2008). Chemical composition and antifungal activity of rosemary (*Rosmarinus officinalis* L.) oil from Turkey. Int. J. Food Sci. Nutr..

[14] Sienkiewicz M., Łysakowska M., Pastuszka M., Bienias W., Kowalczyk E. (2013). The potential of use basil and rosemary essential oils as effective antibacterial agents. Molecules.

[15] Zheljazkov V.D., Astatkie T., Zhalnov I., Georgieva T.D. (2015). Method for attaining rosemary essential oil with differential composition from dried or fresh material. J. Oleo Sci..

[16] Conde-Hernández L.A., Espinosa-Victoria J.R., Trejo A., Guerrero-Beltrán J.Á. (2017). CO_2_-supercritical extraction, hydrodistillation and steam distillation of essential oil of rosemary (*Rosmarinus officinalis*). J. Food Eng..

[17] Boutekedjiret C., Bentahar F., Belabbes R., Bessiere J.M. (2003). Extraction of rosemary essential oil by steam distillation and hydrodistillation. Flavour Fragr. J..

[18] Lo Presti M., Ragusa S., Trozzi A., Dugo P., Visinoni F., Fazio A., Dugo G., Mondello L. (2005). A comparison between different techniques for the isolation of rosemary essential oil. J. Sep. Sci..

[19] Reverchon E., Senatore F. (1992). Isolation of rosemary oil: Comparison between hydrodistillation and supercritical CO_2_ extraction. Flavour Fragr. J..

[20] Müller J., Heindl A. (2006). Drying of medicinal plants. Frontis.

[21] Rocha R.P., Melo E.C. (2011). Influence of drying process on the quality of medicinal plants: A review. J. Med. Plants Res..

[22] Venskutonis P.R. (1997). Effect of drying on the volatile constituents of thyme (*Thymus vulgaris* L.) and sage (*Salvia officinalis* L.). Food Chem..

[23] Piga A., Usai M., Marchetti M., Foddai M., Del Caro A., Meier P., Onorati V., Vinci F. Influence of different drying parameters on the composition of volatile compounds of thyme and rosemary cultivated in Sardinia. Proceedings of the 3rd International Symposium on Food and Agricultural Products: Processing and Innovations.

[24] Hossain M.B., Barry-Ryan C., Martin-Diana A.B., Brunton N.P. (2010). Effect of drying method on the antioxidant capacity of six Lamiaceae herbs. Food Chem..

[25] RAO L.J., Singh M., Raghavan B., Abraham K.O. (1998). Rosemary (*Rosmarinus officinalis* L.): Impact of drying on its flavor quality. J. Food Qual..

[26] Usai M., Marchetti M., Foddai M., Del Caro A., Desogus R., Sanna I., Piga A. (2011). Influence of different stabilizing operations and storage time on the composition of essential oil of thyme (*Thymus officinalis* L.) and rosemary (*Rosmarinus officinalis* L.). LWT-Food Sci. Technol..

[27] Jerković I., Mastelić J., Miloš M. (2001). The impact of both the season of collection and drying on the volatile constituents of *Origanum vulgare* L. ssp. hirtum grown wild in Croatia. Int. J. Food Sci. Technol..

[28] Sefidkon F., Abbasi K., Khaniki G.B. (2006). Influence of drying and extraction methods on yield and chemical composition of the essential oil of *Satureja hortensis*. Food Chem..

[29] Sellami I.H., Wannes W.A., Bettaieb I., Berrima S., Chahed T., Marzouk B., Limam F. (2011). Qualitative and quantitative changes in the essential oil of *Laurus nobilis* L. leaves as affected by different drying methods. Food Chem..

[30] Croteau R., Felton M., Karp F., Kjonaas R. (1981). Relationship of camphor biosynthesis to leaf development in sage (*Salvia officinalis*). Plant Physiol..

[31] Ishihara K., Hamada H., Hirata T., Nakajima N. (2003). Biotransformation using plant cultured cells. J. Mol. Catal. B Enzym..

[32] El-Ghorab A.H. (2003). Supercritical fluid extraction of the Egyptian rosemary (*Rosmarinus officinalis*) leaves and *Nigella sativa* L. seeds volatile oils and their antioxidant activities. J. Essent. Oil Bear. Plants.

[33] Carvalho R.N., Moura L.S., Rosa P.T.V., Meireles M.A.A. (2005). Supercritical fluid extraction from rosemary (*Rosmarinus officinalis*): Kinetic data, extract’s global yield, composition, and antioxidant activity. J. Supercrit. Fluids.

[34] Carrillo J.D., Tena M.T. (2006). Determination of volatile compounds in antioxidant rosemary extracts by multiple headspace solid-phase microextraction and gas chromatography. Flavour Fragr. J..

[35] Amorati R., Foti M.C., Valgimigli L. (2013). Antioxidant activity of essential oils. J. Agric. Food Chem..

[36] Roginsky V., Lissi E.A. (2005). Review of methods to determine chain-breaking antioxidant activity in food. Food Chem..

[37] Mohammed H.A., Abdel-Aziz M.M., Hegazy M.M. (2019). Anti-Oral Pathogens of *Tecoma stans* (L.) and *Cassia javanica* (L.) Flower Volatile Oils in Comparison with Chlorhexidine in Accordance with Their Folk Medicinal Uses. Medicina.

[38] Mohammed H.A., Al-Omar M.S., Aly M.S.A., Hegazy M.M. (2019). Essential Oil Constituents and Biological Activities of the Halophytic Plants, *Suaeda Vermiculata* Forssk and Salsola *Cyclophylla Bakera* Growing in Saudi Arabia. J. Essent. Oil Bear. Plants.

[39] Parejo I., Codina C., Petrakis C., Kefalas P. (2000). Evaluation of scavenging activity assessed by Co (II)/EDTA-induced luminol chemiluminescence and DPPH·(2, 2-diphenyl-1-picrylhydrazyl) free radical assay. J. Pharmacol. Toxicol. Methods.

[40] Nile S.H., Khobragade C.N. (2010). Antioxidant activity and flavonoid derivatives of *Plumbago zeylanica*. J. Nat. Prod..

