# Supplementary material for: Drying Induced Impact on Composition and Oil Quality of Rosemary Herb, Rosmarinus Officinalis Linn
Source: Molecules. 2020 Jun 19;25(12):2830. doi: 10.3390/molecules25122830 (PMC7355710; doi:10.3390/molecules25122830)
Supplement: Supplementary file 1 [file molecules-25-02830-s001.pdf]

Drying induced impact on composition and oil quality of rosemary herb, *Rosmarinus Officinalis* Linn.

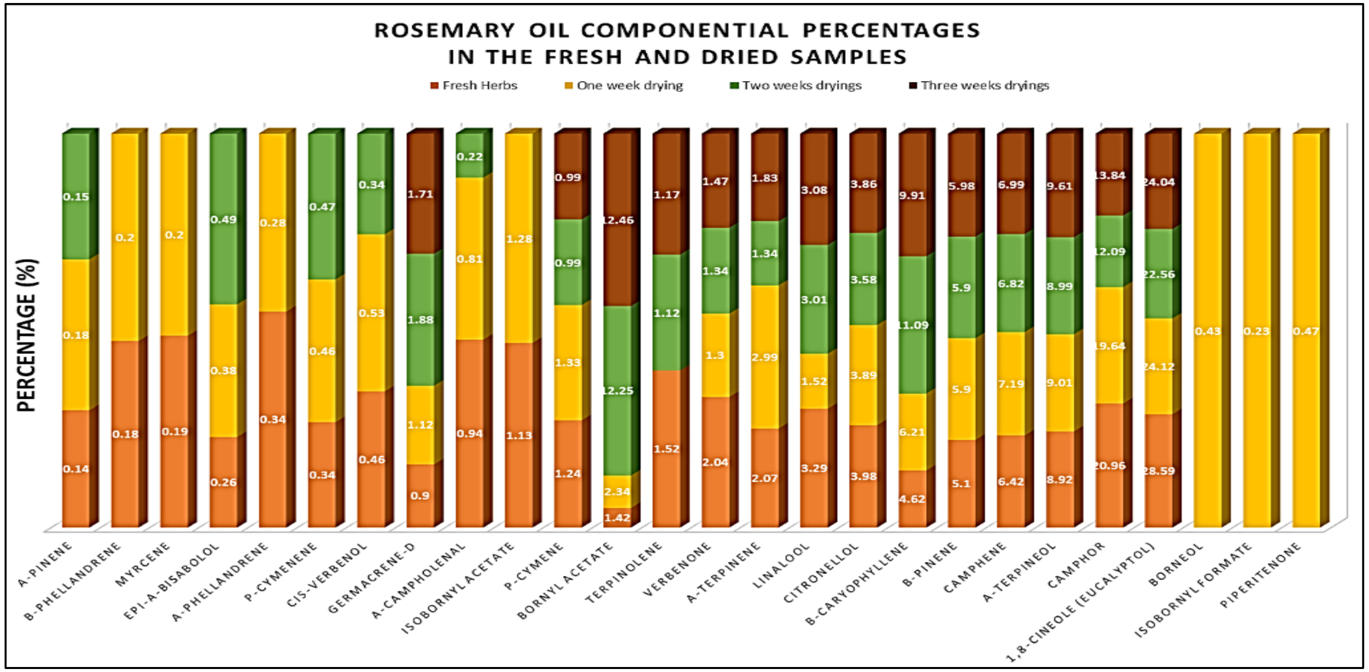

Figure S1: Rosemary oils' componential percentages in fresh and dried samples
